# Separation and purification of antioxidant peptides from purple speckled kidney bean by macroporous adsorption resin and analysis of amino acid composition

**DOI:** 10.3389/fnut.2022.1001456

**Published:** 2022-11-11

**Authors:** Dan Li, Xin-yu Xu, Yang Yang, Na Wu, Zhan-qian Ma, Feng Zuo, Na Zhang

**Affiliations:** ^1^School of Food Science, Heilongjiang Bayi Agriculture University, Daqing, China; ^2^National Cereal Engineering Technology Research Center, Heilongjiang Bayi Agriculture University, Daqing, China; ^3^School of Food Engineering, Harbin University of Commerce, Harbin, China

**Keywords:** purple speckled kidney bean protein, antioxidant peptide, separation, purification, macroporous resin

## Abstract

The protein hydrolysate of purple speckled kidney bean (PSKB) was used as the raw material in this study, and the antioxidant peptide of the PSKB protein hydrolysate was purified using macroporous resin. The XAD-7HP macroporous resin was selected as the best purification material, and the static adsorption-desorption of the purified PSKB antioxidant peptide was optimized. The optimum static adsorption and desorption conditions were as follows: the adsorption capacity reached 11.93 ± 0.11 mg/ml at pH 7 for 24 h, and the desorption capacity was 5.24 ± 0.04 mg/ml with 60% ethanol for 30 min. Under this condition, the amount of antioxidant peptide obtained by adsorption-desorption was the highest. The optimum process conditions were as follows: the appropriate flow rate was 1 ml/min, and the optimal injection volume was 40 ml. The adsorption amount at this time can reach 12.19 ± 0.15 mg/ml. The components with an elution time of 10–30 min were separated using the reversed-phase high-performance liquid chromatography (RP-HPLC) technique to obtain three main components, namely, RP_1_, RP_2_, and RP_3_. The DPPH free radical scavenging ability reached 56.26 ± 0.56, 66.42 ± 0.56, and 78.57 ± 0.56%, respectively, which were 36.65, 46.34 ± 0.56, and 54.39 ± 0.56% higher than those before purification. The amino acid sequences of the three components were identified as Phe-Leu-Val-Asp-Arg-Ile, Phe-Leu-Val-Ala-Pro-Asp-Asp, and Lys-Asp-Arg-Val-Ile-Ser-Glu-Leu.

## Introduction

Purple speckled kidney bean (PSKB), commonly known as *Phaseolus vulgaris* L., is one of the most globally important edible legume crops in humans, and it is rich in components of high amounts of proteins (20–25%), complex carbohydrates (50–60%), and many essential vitamins and minerals (copper, calcium, iron, magnesium, manganese, and zinc) (1). PSKB protein peptide is an oligopeptide, which is an intermediate product between proteins and amino acids. In special food applications, protein hydrolysates have been used as high value-added ingredients, with high protein digestibility and amino acid selection. This type of food is indispensable for people with chronic conditions such as Crohn's disease, who have difficulty absorbing the protein in its natural form. Studies found that small-molecule peptides have good biological activity and strong antioxidant properties (2).

To improve the value of PSKB, protein hydrolysates were separated from the protein. Using PSKB albumin as raw material, Hongxiu Fan et al. found that, after enzymatic hydrolysis, the antioxidant activity, foaming properties, and PSKB albumin stability were significantly improved, and ultraviolet absorption was enhanced (3). Using PSKB peptide as a test material, Mehnaza Manzoor et al. found that the protein peptide can reduce the oxidative damage of HepG_2_ cells caused by H_2_O_2_ to a certain extent, can also regulate the cell's redox system, remove intracellular reactive oxygen species, and improve intracellular antioxidant enzyme activity (4). Macroporous adsorption resin was developed in the 1960s and was used as a new type of non-ionic porous polymer adsorbent. Its separation characteristics are adsorption and the molecular sieve principle (5). At present, macroporous resins are widely used in the separation and purification of active ingredients such as total flavonoids, polyphenols, anthocyanins, polysaccharides, alkaloids, and saponins (6–11). Tingting Bu et al. (12) used macroporous adsorption resin to adsorb the small molecular peptide in whey protein hydrolysate and found that the percentage of peptide below 600 Da was 93.60%, which was 42.81% higher than that before adsorption. The adsorption effect was significant. Macroporous adsorption resin has potential application prospects in the field of protein-peptide adsorption. Using different types of macroporous resins, Cerrone Cabanos et al. (13) compared the adsorption effects of alfalfa leaf protein peptides, screened the best macroporous adsorption resin as DA201-C, and studied its adsorption-desorption characteristics for alfalfa leaf protein peptides. Mingzhu Zhuang et al. (14) explored the adsorption and desorption performances of 4 medium-sized macroporous adsorption resins on soybean peptides. The results showed that different resins had significant differences in the purification of raw materials and the enrichment of peptides. The non-polar resins with large specific surface area and small pore size had good adsorption and desorption properties.

At present, there are many studies on the physicochemical properties and single-enzyme hydrolysis technology of PSKB protein. However, the amino acid sequence of PSKB is less studied. At the same time, the amino acid sequence also limits the production, processing, and application of the antioxidant peptides from PSKB. So, in this study, the PSKB antioxidant peptides were prepared by simultaneous hydrolysis of complex protease, the static adsorption and desorption conditions were optimized, and the structure was identified, which laid a certain research foundation for the deep processing of PSKB protein and for the development and utilization of PSKB peptides.

## Materials and methods

### Materials

The purple speckled kidney bean, which was produced in Heilongjiang Province in China, was provided by the National Cereal Engineering Technology Research Center in Daqing, Heilongjiang Province.

Pepsin (3,000 U/g) and trypsin (74,000 U/g) were purchased from Germany's Saiguo Biotechnology Co., Ltd. The HPD-400, HPD-400A, and XAD-7HP resins were purchased from Cangzhou Baoen Adsorption Material Technology Co., Ltd. Potassium sodium tartrate was purchased from Liaoning Quanrui Reagent Co., Ltd. Anhydrous ethanol was purchased from Shandong Deyan Chemical Co., Ltd. DPPH, ferrous sulfate, and ferrous chloride were of analytical grade and were purchased from Aladdin Reagent Co., Ltd. The PHS.2C precision pH meter was purchased from Mettler Toledo, USA. RP-HPLC was purchased from Agilent, USA.

### Extraction of PSKB protein and preparation of hydrolysate

PSKB protein extraction is based on the experimental methods of Li Yuqiong (15, 16) and so on. The two enzymes with the best hydrolysis effects, neutral protease and alkaline protease, were screened through preliminary experiments. The double-enzyme simultaneous composite hydrolysis experiment was performed. The single-factor combined orthogonal experiment was used to optimize the dual-enzyme simultaneous hydrolysis process. The optimal hydrolysis conditions were as follows: The temperature was 55°C, the pH was 10, the alkaline protease:neutral protease ratio was 1:2, and 5 ml of the mixed enzyme was added. Under this condition, PSKB had a proteolysis degree of 42.2% and had good antioxidant activity.

### Macroporous resin pretreatment

Three resins, HPD-400, HPD-400A, and XAD-7HP, were selected. The adsorption and desorption properties of the three resins were investigated, and the types of macroporous resins suitable for the separation and purification of the antioxidant peptide from PSKB were selected (see Table 1 for details).

**Table 1:** Physical structure parameters of different macroporous resins.

**Type of resin**	**Specific surface area/(m^2^.g)**	**Types of polarity**	**Average pore size/A**
XAD-7HP	500–550	Polarity	75–80
HPD-400	500–550	Polarity	85–90
HPD-400A	480–500	Weak polarity	450–500

### Static adsorption of PSKB proteolysate by macroporous resin

#### Determination of the best resin

A total of 15 g of pretreated resin was added to a 250-ml stoppered Erlenmeyer flask, and 100 ml of PSKB antioxidant peptide solution was added after enzymatic hydrolysis to completely soak the resin. The flask was sealed and shaken at 180 r/min in a constant temperature shaker for 24 h to completely contact the resin with the crude peptide solution. After it was fully adsorbed, 1 ml of the supernatant was absorbed, mixed with 4 ml of the biuret reagent, and measured at 540 nm after 30 min. The remaining peptide concentration in the adsorption solution was used to calculate the resin adsorption (17).


$$\begin{array}{l} \text{Adsorption capacity (mg/g)}=\frac{C-C_{0}}{m}\times V\\ \;\text{Adsorption rate (\%)}=\frac{C-C_{0}}{C}\times 100 \end{array}$$

In the formula, *C* and *C*_0_ are the protein concentrations (mg/ml) before and after adsorption, respectively; *V* is the volume (ml) of the PSKB antioxidant peptide solution.

#### Static adsorption curve

A total of 10 g of screened resin was added to a 250-ml Erlenmeyer flask, 100 ml of PSKB protein hydrolysate was added after concentration treatment, and the bottle mouth was sealed and shaken in a constant temperature shaker (30°C, 180 r/min). The solution was collected every 1 h, the peptide content in the solution was determined after adsorption, the adsorption rate and adsorption amount of PSKB peptide were calculated according to the formula, and a static adsorption curve was drawn.

#### Static desorption and selection of eluent

Static adsorption was performed as described in the “Determination of the best resin” section, shaken in a constant temperature shaker for 12 h, the filter was removed when it was fully adsorbed, and then the adsorbed macroporous resin was placed in a 250-ml conical flask again. After the adsorption of the PSKB antioxidant peptides, the resin was eluted. The eluents were deionized water (0%) and 20, 40, 60, and 80% ethanol solutions of 20 ml each, which were shaken under the same conditions. After 10, 20, 30, 40, and 50 min of desorption, the volume and the protein content of the desorption solution were measured, and a static desorption curve was drawn.


$$\begin{array}{l} \text{Desorption }\left(\text{mg}/\text{mL}\right)=\text{Desorbed protein concentration}\\ \;\times \text{ Desorption volume} \end{array}$$

#### Static adsorption capacity test of PSKB hydrolysate with different Ph values

A total of 10 g of the treated macroporous adsorption resin was weighed, placed in a 250-ml triangle bottle, and 50 ml of PSKB protein hydrolysate with different pH values was added (4.0, 5.0, 6.0, 7.0, 8.0, 9.0, and 10.0) so that the resin was completely soaked and placed in a constant temperature shaker (180 r/min) at 30°C. After adsorption for certain periods of time, it was filtered, and the volume of the hydrolysate and the peptide content before and after the adsorption were used to calculate the amount of adsorption.

### Dynamic adsorption and desorption of PSKB antioxidant peptides by macroporous adsorption resin

We weighed 100 g of the treated XAD-7HP resin into a 1.0 × 100 cm chromatography column, and the PSKB antioxidant peptide solution at pH 7.0 was 1.0, 1.5, and 2.0 ml at 30°C. The column was loaded at a minimum flow rate, the mass concentration of the effluent was checked, and the adsorption curve of the baseline was considered as the transmission point to obtain the optimal loading volume and loading quality. A 60% ethanol solution was used as the eluent (flow rate of 2 ml/min). The eluent was collected at a certain dose every 10 min. The absorbance of the collected solution was measured with a UV detector at a wavelength of 540 nm. The eluted peaks were collected and dried for later use. With reference to the method of Pablo R. Salgado et al. (18), the antioxidative capacity of each component peptide solution was tested at a concentration of 1%. In a stoppered test tube, 0.5 ml of PSKB protein hydrolysate and 3.5 ml 1 × 10^−4^ mol/L DPPH absolute ethanol solution were collected, mixed well, and let stand at room temperature for 30 min. Absolute ethanol was used as a blank control. Using VC as a positive control, the absorbance value was measured at a wavelength of 517 nm, and the DPPH clearance ability was calculated according to the following formula.


$$\begin{array}{l} DPPH\;\text{clearance rate (\%)}=\left(\frac{A_{1}}{A_{2}-A_{3}}\right)\times 100\%, \end{array}$$

where *A*_1_ is the absorbance value of the DPPH solution after adding the PSKB protein hydrolysate; *A*_2_ is the absorbance value of the PSKB protein hydrolysate solution; *A*_3_ is the absorbance value of DPPH solution without adding the PSKB protein hydrolysate.

### RP-HPLC for the detection of antioxidant peptides in PSKB

The hydrolysate and the purified antioxidant peptide of PSKB protein were, respectively, treated by reversed-phase high-performance liquid chromatography, and the components and antioxidant capacity of each component were analyzed. The experimental conditions were as follows: Agilent C18; mobile phase: acetonitrile:water:trifluoroacetic acid: 20:80:0.02%); flowrate: 1.0 ml/min; column temperature: 25°C; ultraviolet detection; and detection wavelength: 540 nm.

### Amino acid sequence determination of antioxidant peptides from PSKB

The antioxidant peptide of PSKB was isolated from macroporous resin for the identification of amino acid sequence, and the method was adapted from Jiaqi Fang et al. (19, 20) and modified appropriately. Chromatographic conditions were as follows: mobile phase A (mass spectrometric ultrapure water containing 0.1% formic acid), mobile phase B (mass spectrometric acetonitrile containing 0.1% formic acid), a flow rate of 200 μl/min (2 μl/min after shunt), and a gradient of 120 min (5% B 15 min, 5–32% B 45 min, 90% B 35 min, 5% B 5 min, 5% B equilibrium 20 min). The mass spectrometry of electrospray operating voltage was 3.5 kV; the ion migration tube temperature was 250°C; fragments of the spectrum diagram to make the tandem mass spectrometry (MS), according to the same energy cracking, was set to collision-induced dissociation (collisionally induced dissociation, CID); collision energy was 35%; the ionization method was electrospray ionization (ESI); the scanning range, in terms of mass-to-charge ratio (m/z), was 400–1,800. The secondary mass spectrum retrieval software uses Proteome Discovery 1.2, and the retrieval algorithm is a sequence.

### Statistical analysis

All experiments were done in triplicates and were expressed as means ± SD. Data were analyzed using a one-way analysis of variance (ANOVA) using SPSS statistics 20.0 (SPSS Inc, USA). The significant differences were determined using Duncan's multiple range tests at a *P*-value of < 0.05.

## Results and analysis

### Screening of macroporous adsorption resin

**Figure 1:**
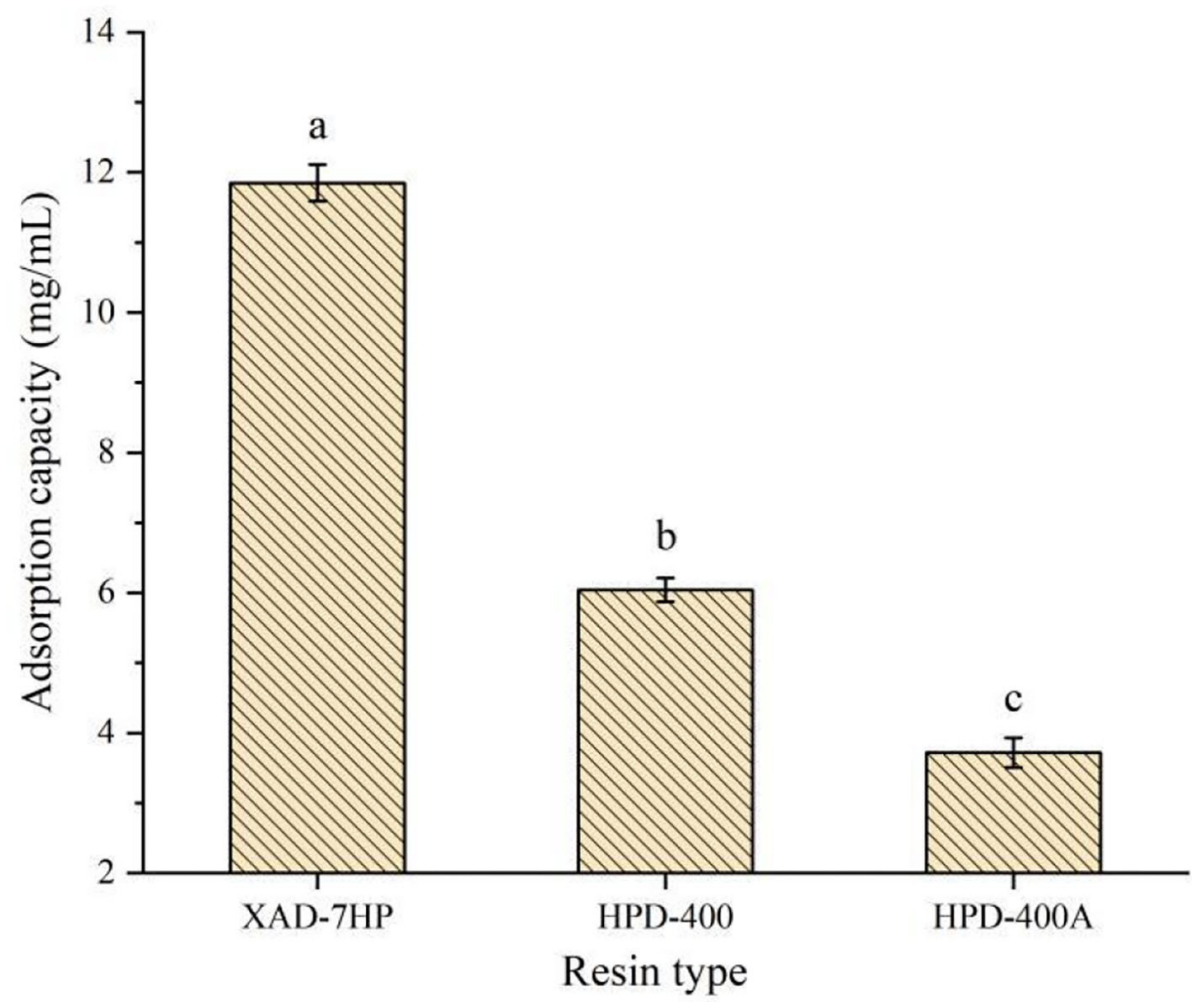
Adsorption capacity of different types of resins to protein peptides of PSKB. There is a significant difference between different small letters (*P* < 0.05).

Due to the different specific surface areas, surface electrical properties, and hydrophobic interaction with adsorbed substances, different resins have a significant impact on the adsorption capacity of antioxidant peptides in PSKB (21, 22). The structural parameters of the three macroporous adsorption resins selected in the test are shown in Table 1. Table 1 shows that XAD-7PH and HPD-400 are polar resins with small pore diameters; HPD-400A is a weak polar resin with a large pore size. The adsorption capacity of antioxidant peptides in PSKB by different types of resins is shown in Figure 1. It can be seen from Figure 1 that XAD-7PH polar resin with the smallest pore size has the strongest adsorption capacity, which is 11.85 ± 0.26 mg/ml. With the increase in pore size, the adsorption capacity of macroporous adsorption resin gradually decreased, in which HPD-400 resin was 6.04 ± 0.17 mg/ml and HPD-400A resin was 3.72 ± 0.21 mg/ml. The adsorption capacities of the three resins were significantly different (*P* < 0.05). XAD-7PH polar resin with the smallest pore size has better adsorption capacity for antioxidant peptides, which mainly depends on dipole ions and hydrogen bonds in the molecule (23). Therefore, we used XAD-7HP resin to separate antioxidant peptides from PSKB.

### Study on static adsorption of protease hydrolysate from PSKB by macroporous resin

#### Static adsorption curves of PSKB protein hydrolysates at different pH values

**Figure 2:**
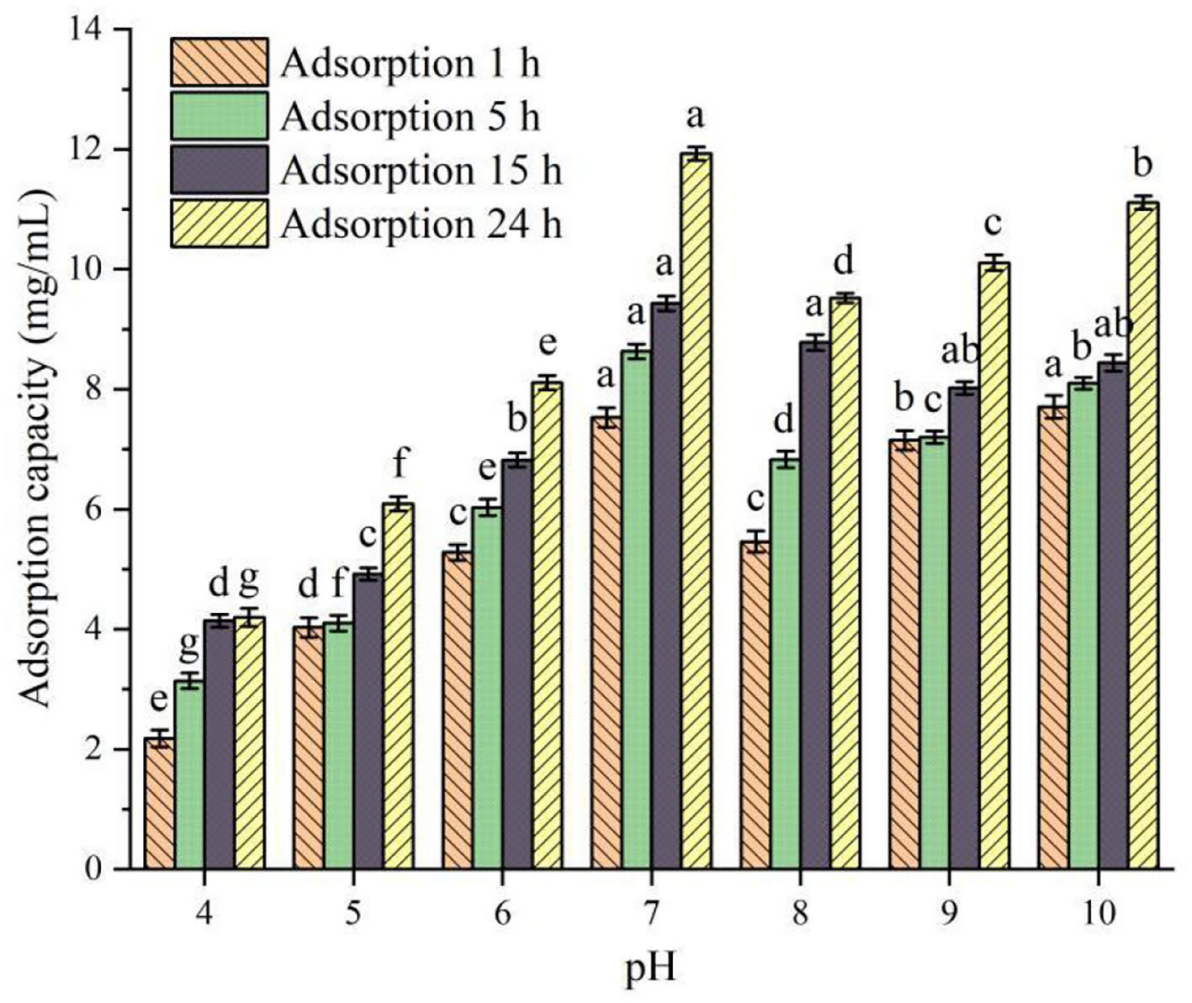
Adsorption capacity of PSKB protein hydrolysate at different pH values. There is a significant difference between different small letters (*P* < 0.05).

The relationship between the static adsorption capacity of XAD-7HP macroporous adsorption resin and pH value is shown in Figure 2. It can be seen from Figure 2 that, under the same adsorption time, the adsorption capacity of XAD-7HP resin under different pH conditions for PSKB protein hydrolysates is significantly different (*P* < 0.05). Among them, with the increase in pH, the adsorption capacity of XAD-7HP resin on PSKB protein hydrolysates shows an upward trend, reaching the maximum at pH = 7, which is 7.53–11.93 mg/ml. With the continuous increase in pH, the adsorption capacity of XAD-7HP resin on PSKB protein hydrolysates slightly decreases as the adsorption capacity of the resin is related to van der Waals force and hydrogen bond, and its network structure and specific surface area have certain screening performance (24). Under the same pH condition, the adsorption capacity of XAD-7HP resin to PSKB protein hydrolysate was gradually enhanced with the extension of adsorption time (*P* < 0.05). At pH = 7 and an adsorption time of 24 h, the adsorption capacity was the strongest, reaching 11.93 ± 0.11 mg/ml.

#### Static desorption experiment and selection of desorption agent

**Figure 3:**
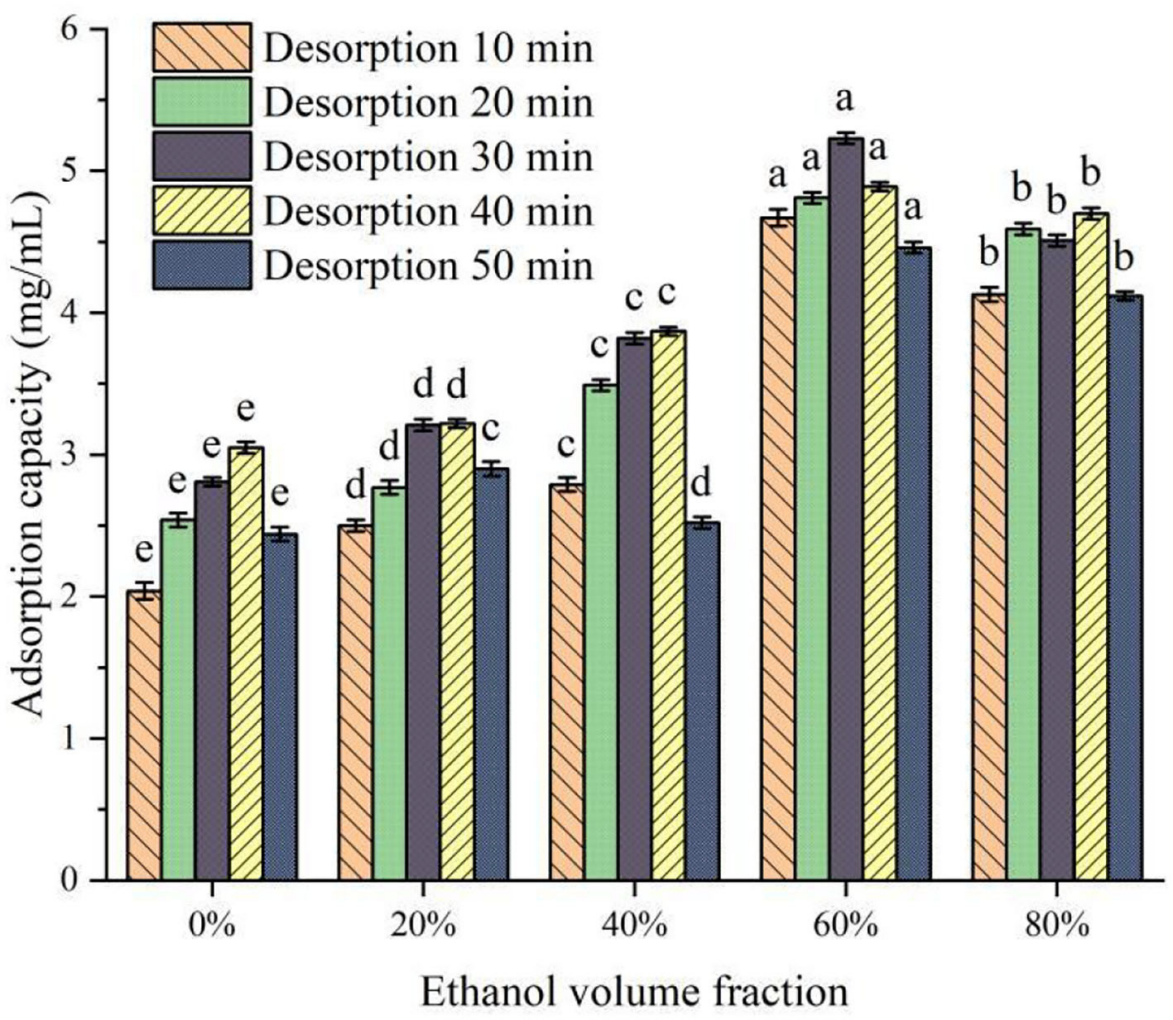
Effect of ethanol solution with different volume fractions on desorption. There is a significant difference between different small letters (*P* < 0.05).

The molecular polarity of ethanol solution with different volume fractions is different, and the binding ability with protein peptides and the adsorption ability of covalent bonds are different (25). The change in desorption capacity of XAD-7HP macroporous adsorption resin with different ethanol volume fractions is shown in Figure 3. It can be seen from Figure 3 that the desorption ability of desorbers with different ethanol volume fractions on PSKB protein hydrolysate is significantly different (*P* < 0.05). With the increase in ethanol volume fraction, the desorption capacity of the desorber to PSKB protein hydrolysate showed an overall upward trend, reaching the highest when the ethanol volume fraction was 60%, which was 4.46–5.23 mg/ml. The volume fraction of ethanol continued to increase, and the desorption capacity slightly decreased. When the volume fraction of ethanol was 80%, it was 4.12–4.70 mg/ml. This is because the volume fraction of ethanol is too high, which makes it difficult for proteins and peptides to dissolve. With the prolongation of desorption time, the desorption amount of protein hydrolysate by the desorber increased first and then decreased. When the desorption time reached 30 min, the desorption amount reached the maximum. This is mainly because, with the desorption time extension, the probability of contact between the desorption solution and the protein peptide of PSKB adsorbed on the resin is increased, more easily elution PSKB polypeptide from the resin. However, with the increase in desorption time, the resin reached saturation resolution, and its resolution did not increase with time. Therefore, the volume fraction of ethanol in the desorption solution was 60%, and the desorption for 30 min was a suitable analytical condition, at which time the desorption volume reached 5.23 ± 0.04 mg/ml.

### Dynamic adsorption and desorption of macroporous resin

#### Selection of the best feeding speed and quantity

**Figure 4:**
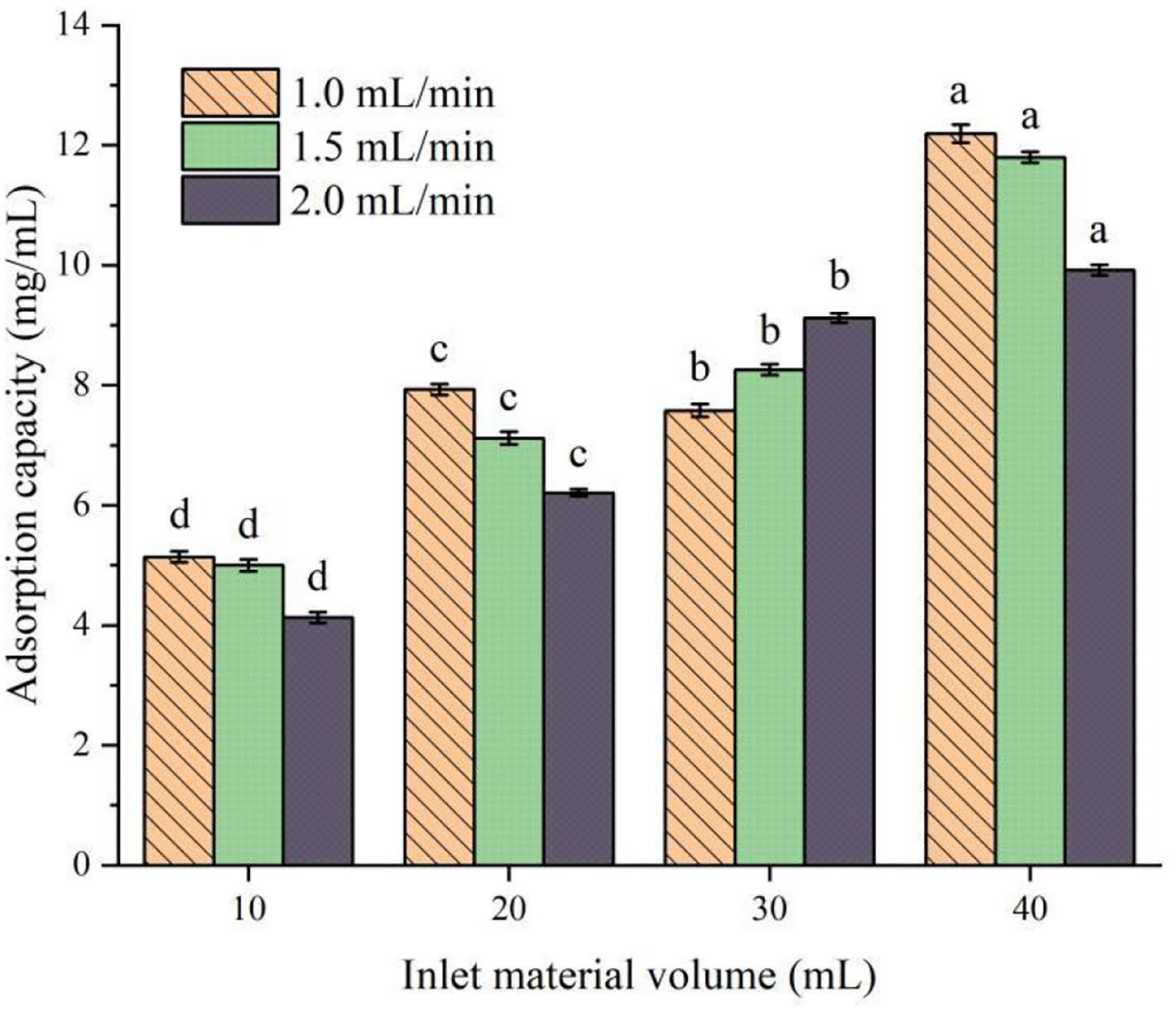
Effects of different flow rates and feed amounts on the adsorption of protein peptides in PSKB. There is a significant difference between different small letters (*P* < 0.05).

The effect of different flow rates and feeding amounts on the adsorption of protein peptides in speckled kidney beans is shown in Figure 4. It can be seen from Figure 4 that, when the control flow rate is the same and the feed amount is different, there is a significant difference among the components (*P* < 0.05). With the increase in feed amount, the adsorption capacity of XAD-7HP resin to PSKB protein peptide increases with the increase in PSKB and reaches the maximum when the feed amount is 40 ml, which is 9.92–12.19 mg/ml. Under the same feeding amount, the increase in the flow rate decreases the adsorption capacity of PSKB protein peptide, and the adsorption capacity decreases significantly when the flow rate is greater than 1.5 ml/min (*P* < 0.05). This is mainly because the feed flow rate directly determines the diffusion degree and binding law of the adsorbed substance to the resin. Although the sample can fully contact the resin when the flow rate is too low, it will prolong the industrial treatment time and increase production cost, and if the flow rate is too high, the adsorbed substance will be washed down before it combines with the resin in time, resulting in lower adsorption (26, 27). Therefore, the loading flow rate of 1 ml/min was selected as the appropriate flow rate, and the feeding volume of 40 ml was the optimal injection volume. At this point, the adsorption volume could reach 12.19 ± 0.15 mg/ml.

#### Dynamic desorption test of macroporous resin

**Figure 5:**
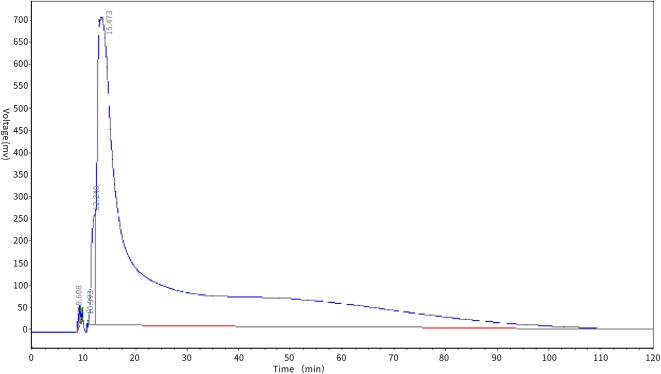
Antioxidation peptide desorption test of PSKB.

The desorption effect of XAD-7HP macroporous adsorption resin on antioxidant peptides in PSKB is shown in Figure 5. It can be seen from Figure 5 that the protein peptide of PSKB began to be eluted at about 10 min, and then, the voltage value of the desorption curve rose rapidly and then dropped sharply, which may be due to the high content of PSKB protein peptide on the resin before desorption. At 25 min, the desorption curve began to show a flat state and the elution rate slowed down. This is mainly because the remaining polypeptide is mainly composed of relatively high molecular weight components. The components with relatively high molecular weight are less soluble in 60% ethanol solution and are not easily desorbed. Therefore, the desorption curve is relatively stable in the latter stage (28). With the extension of elution time, the content of protein peptide in the resin decreases, and the PSKB protein peptide adsorbed is desorbed at approximately 60 min, which indicates that XAD-7HP macroporous adsorption resin can adsorb PSKB antioxidant peptides, and the desorption is easier.

#### DPPH clearance rates of PSKB protein peptides with different desorption fluids

**Figure 6:**
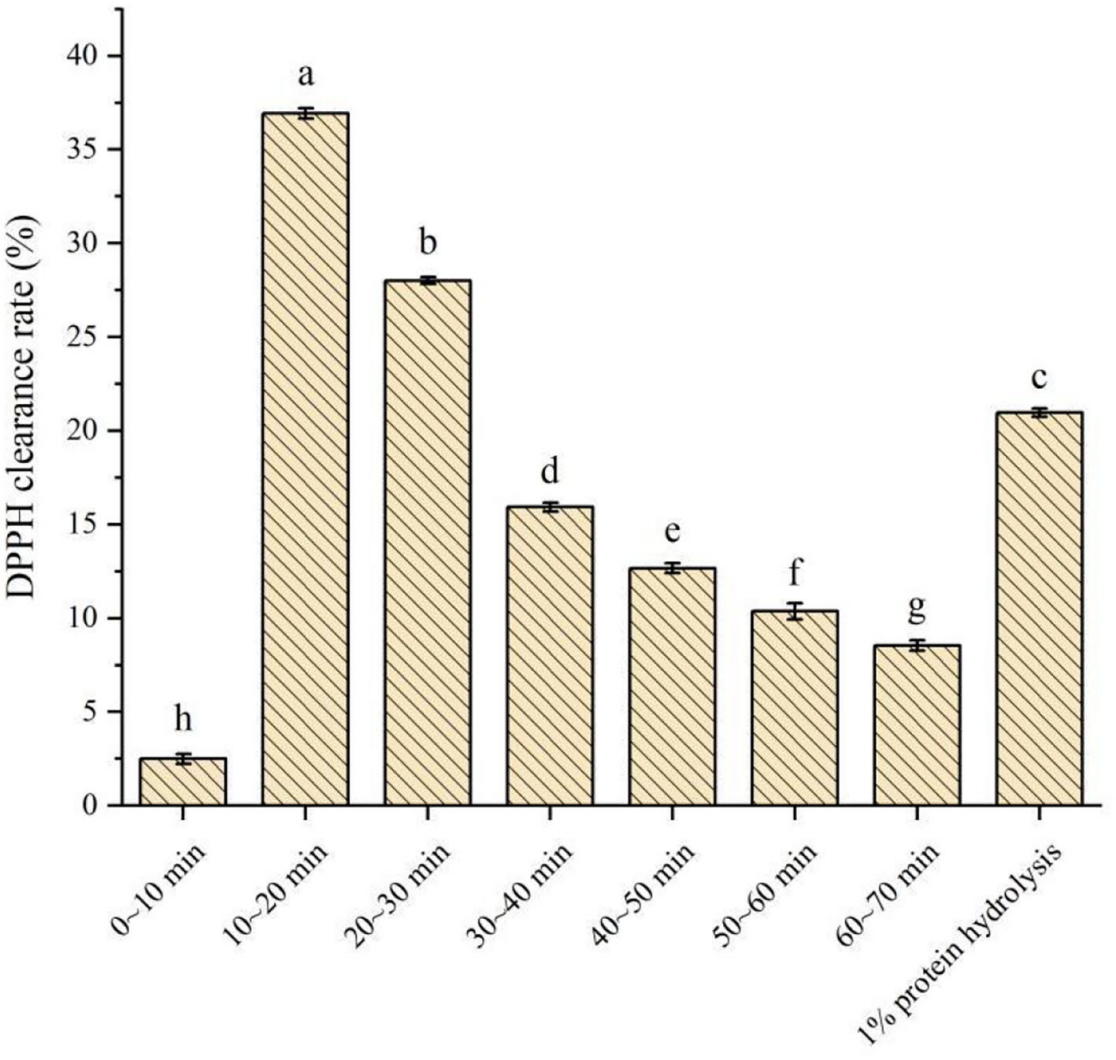
Determination of antioxidant capacity of purified components of PSKB protein peptide. There is a significant difference between different small letters (*P* < 0.05).

The DPPH clearance rates of different desorption fluids of PSKB protein peptides are shown in Figure 6. It can be seen from Figure 6 that the DPPH clearance rate of PSKB antioxidant peptide samples at different time periods was significantly different (*P* < 0.05). Among them, the antioxidant activity was very low at 0–10 min, and the DPPH clearance rate was only 2.5 ± 0.26%, indicating that the PSKB peptide with strong antioxidant activity was not removed at the first 10 min, which was consistent with the dynamic desorption results above. From 10 min, the collected liquid has certain activity, and the activity is concentrated in 10–30 min. Among them, the DPPH clearance rate of 10–20 min desorption solution was 36.93 ± 0.27% and that of 20–30 min desorption solution was 28.02 ± 0.18%. With the prolongation of the desorption time, the DPPH clearance rate of the desorbed solution gradually decreased, and the lowest was 8.54 ± 0.28% at 60–70 min. The DPPH clearance of 1% unpurified PSKB protein hydrolysate was 20.97 ± 0.22%, which was significantly lower than the antioxidant activity of PSKB peptides collected in 10–30 min (*P* < 0.05). This indicated that the antioxidant peptides of PSKB after purification by XAD-7HP resin were concentrated in the components with an elution time of 10–30 min when the antioxidant capacity of PSKB polypeptide was the highest.

### Characterization of antioxidant peptide in PSKB

#### RP-HPLC analysis

**Figure 7:**
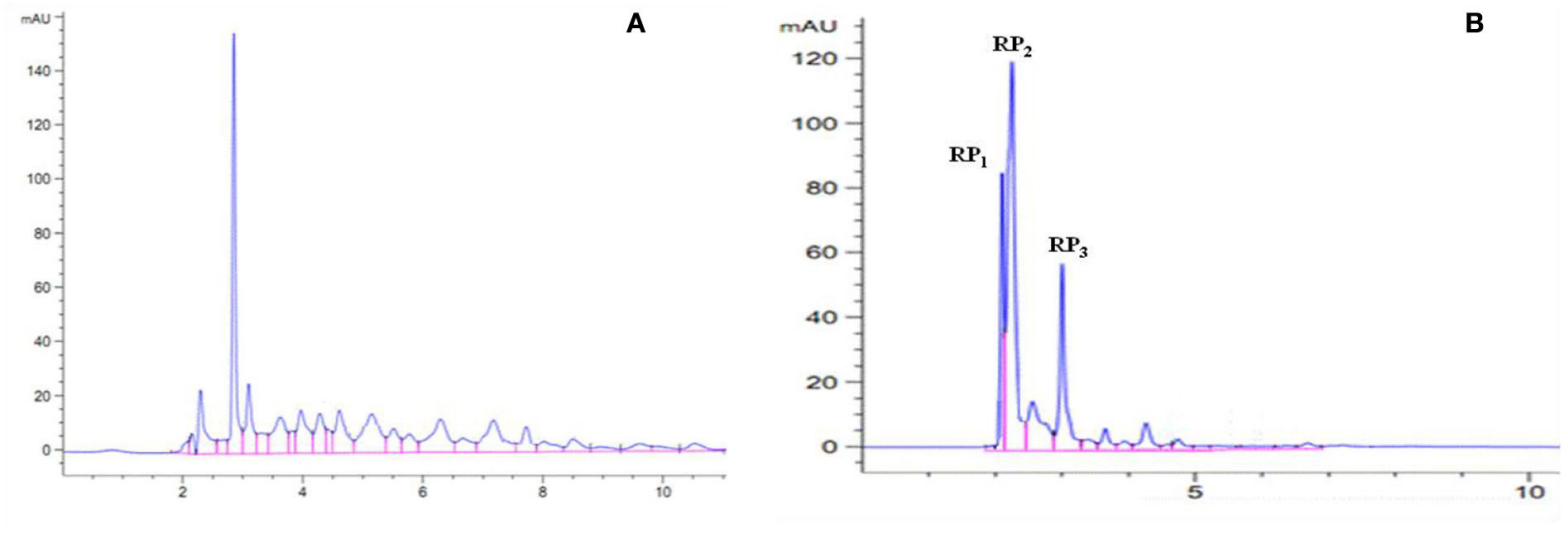
Composition of PSKB protein hydrolysate. **(A)** The composition of the unpurified PSKB antioxidant peptide. **(B)** The composition of PSKB antioxidant peptides purified by XAD-7HP resin.

After the enzymatic hydrolysis of proteins, peptides with different molecular weights were produced. The length of the peptide chain was closely related to the biological activity of peptides (29, 30). The wheat embryo polypeptide with a molecular weight of 180–2,000 Da has stronger antioxidant activity than the macromolecular polypeptide (31). Therefore, the PSKB protein hydrolysate was further analyzed using RP-HPLC in this study. The composition of PSKB protein hydrolysate is shown in Figure 7. Figure 7A shows the composition of the unpurified PSKB antioxidant peptides. It can be seen from Figure 7A that the components of the purified PSKB antioxidant peptide are relatively complex, and the peaks are dispersed in 2–12 min; Figure 7B shows the composition of PSKB antioxidant peptides purified by XAD-7HP resin. The peaks are concentrated in 2–7 min, with few miscellaneous peaks. The three most important peaks are RP_1_, RP_2_, and RP_3_.

The purified PSKB antioxidant peptide was prepared into 1% protein solution, and the antioxidant activity of each component of the sample before and after purification was characterized, as shown in Table 2. It can be seen from Table 2 that there was a significant difference in the DPPH clearance rate of PSKB protein peptide before and after purification (*P* < 0.05). Among them, the DPPH clearance rate of PSKB protein peptide before purification was 35.64 ± 0.56%, and the DPPH clearance rate of PSKB protein peptide after purification was 64.32 ± 0.56%. After purification, there was also a significant difference in DPPH clearance among the components (*P* < 0.05). Among them, RP_3_ had the highest antioxidant activity, and the DPPH clearance capacity was 78.57 ± 0.56%.

**Table 2:** Antioxidant peptide scavenging DPPH clearance rate before and after separation.

**Sample**	**DPPH Clearance rate/%**
Before the separation	35.64 ± 0.56^d^
After the separation	64.32 ± 0.56^bc^
RP_1_	56.26 ± 0.56^b^
RP_2_	66.42 ± 0.56^c^
RP_3_	78.57 ± 0.56^a^

There is a significant difference between different small letters (*P* < 0.05).

#### Amino acid sequencing of antioxidant peptides in PSKB

**Figure 8:**
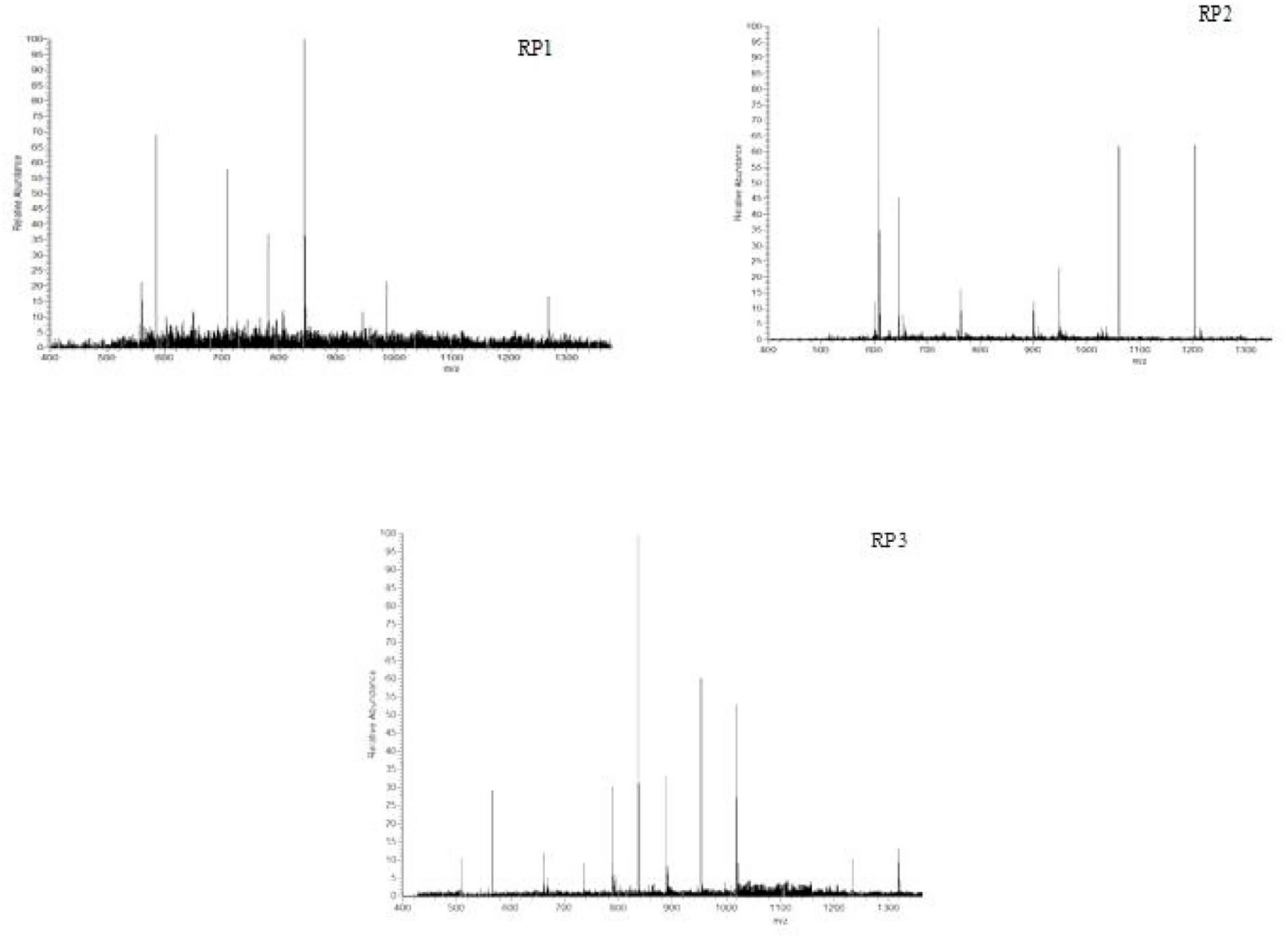
First-order structure mass spectrum of PSKB antioxidant peptides.

Mass spectrometry is a method for the separation and determination of samples according to the mass-to-charge ratio of ions (*m*/*z*), which can be qualitative and quantitative analysis (32–34), and has a high accuracy of qualitative analysis and quantitative analysis of the characteristics of the reliability. The mass spectrogram of antioxidant peptides RP_1_, RP_2_, and RP_3_ in PSKB is shown in Figure 8. The composition of antioxidant peptides was complex and consisted of a variety of peptides. Among them, the RP_1_ showed that it consisted of 6 amino acid residues, the amino acid sequence was Phe-Leu-Val-Asp-Arg-Ile (confirm whether these amino acids are PSKB protein amino acids, the same as below) (Figure 8 RP_1_), and the relative mass was 885.1; RP_2_ was made up of 7 amino acid residues, the amino acid sequence was Phe-Leu-Val-Ala-Pro-Asp-Asp (Figure 8 RP_2_), and the relative mass is 961.06; RP_3_ was composed of 8 amino acid residues, the amino acid sequence is Lys-Asp- Arg-Val-Ile-Ser-Glu-Leu (Figure 8 RP_3_), and the relative mass was 1,043.13.

## Conclusion

In this study, PSKB protein hydrolysate was purified by macroporous adsorption resin, and suitable adsorption resin and adsorption conditions were selected. The results showed that XAD-7HP resin had a strong adsorption capacity, and the adsorption capacity was the strongest at pH 7, adsorption time was 24 h, the sample flow rate was 1 ml/min, and the feed rate was 40 ml. Desorption in 60% ethanol solution for 30 min was the best. The elution time of the enriched antioxidant peptide was concentrated at 10–30 min. Further purification by RP-HPLC yielded three antioxidants with high components, of which RP_3_ had the strongest antioxidant activity, and the DPPH scavenging capacity reached 78.57 ± 0.56%. The amino acid sequence was Lys-Asp-Arg-Val-Ile-Ser-Glu-Leu. This study provides a theoretical basis for the deep processing of PSKB and the development of antioxidant peptide drugs.

## Data availability statement

The raw data supporting the conclusions of this article will be made available by the authors, without undue reservation.

## Author contributions

Conceptualization: NZ and FZ. Writing—original draft preparation: DL. Writing—review and editing: YY. Data curation: X-yX and NW. Formal analysis: Z-qM. Methodology: X-yX. All authors contributed to the article and approved the submitted version.

## Conflict of interest

The authors declare that the research was conducted in the absence of any commercial or financial relationships that could be construed as a potential conflict of interest.

## Publisher's note

All claims expressed in this article are solely those of the authors and do not necessarily represent those of their affiliated organizations, or those of the publisher, the editors and the reviewers. Any product that may be evaluated in this article, or claim that may be made by its manufacturer, is not guaranteed or endorsed by the publisher.
